# Supplementary material for: Phenotype of circulating tumor-reactive T cells predicts immune checkpoint inhibitor response in non-small cell lung cancer
Source: Nat Commun. 2026 Feb 17;17:2856. doi: 10.1038/s41467-026-69680-x (PMC13022356; doi:10.1038/s41467-026-69680-x)
Supplement: Supplementary file 1 — Supplementary Information [file 41467_2026_69680_MOESM1_ESM.pdf]

Supplementary Information for

Phenotype of Circulating Tumor-Reactive T Cells Predicts Immune Checkpoint Inhibitor Response in Non-Small Cell Lung Cancer

Katsuhiro Ito[1,2], Kei Iida[3,4], Tomoko Hirano[1], Merrin Man Long Leong[1], Kenji Morii[1], Toshi Menju[5], Hiroshi Date[5], Hiroaki Ozasa[6], Hironori Yoshida[6], Toyohiro Hirai[6], Shusuke Kawashima[7], Kazuhiro Aoyama[7], Yuka Saeki[7], Takashi Inozume[7], Takashi Kobayashi[2], Kenji Chamoto[1,8*], Tomonori Yaguchi[1,8,9*]

1. Department of Immunology and Genomic Medicine, Center for Cancer Immunotherapy and Immunobiology, Graduate School of Medicine, Kyoto University; Kyoto, Japan.

2. Department of Urology, Graduate School of Medicine, Kyoto University; Kyoto, Japan.

3. Infomatics Platform, Center for Cancer Immunotherapy and Immunobiology, Graduate School of Medicine, Kyoto University; Kyoto, Japan.

4. Faculty of Science and Engineering, Kindai University; Osaka, Japan.

5. Department of Thoracic Surgery, Graduate School of Medicine, Kyoto University; Kyoto, Japan.

6. Department of Respiratory Medicine, Graduate School of Medicine, Kyoto University; Kyoto, Japan.

7. Department of Dermatology, Chiba University Graduate School of Medicine; Chiba, Japan.

8. Department of Immuno-oncology PDT, Graduate School of Medicine, Kyoto University; Kyoto, Japan.

9. Department of Immune Metabolism, Center for Cancer Immunotherapy and Immunobiology, Graduate School of Medicine, Kyoto University; Kyoto, Japan.

*Corresponding: Tomonori Yaguchi and Kenji Chamoto

Division of Clinical Immunology and Cancer Immunotherapy, Center for Cancer Immunotherapy and Immunobiology, Graduate School of Medicine, Kyoto University, Yoshida-Konoe-cho, Sakyo-ku, Kyoto 606-8315, Japan.

Tomonori Yaguchi: yaguchi.tomonori.4m@kyoto-u.ac.jp; Tel. +81-75-753-4371

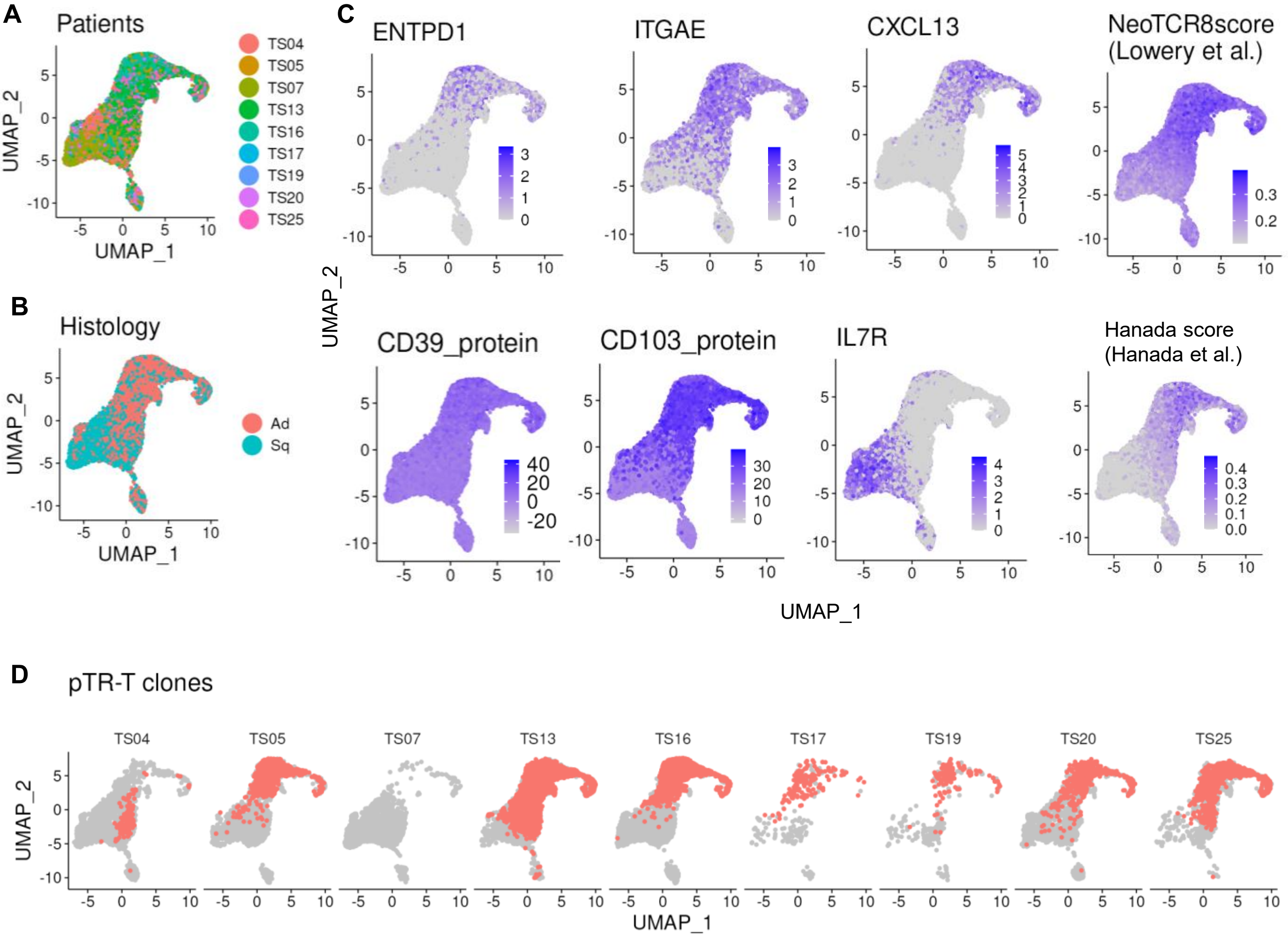

Supplementary Figure 1. Detection of TR-Ts from high-resolution single-cell analysis
(A) UMAP plot of $CD8^+$ TILs from patients with NSCLC group by patients and (B) histological subtype (Ad: adenocarcinoma; Sq: squamous cell carcinoma).
(C) Expression of previously reported marker genes (*ENTPD1*, *CXCL13*, and *ITGAE*), marker proteins (CD39 and CD103), and gene signatures (Lowery et al [23] and Hanada et al [26]) predictive of tumor-reactive T cells (pTR-Ts). *ENTPD1* encodes CD39, and *ITGAE* encodes CD103.
(D) Distribution of pTR-Ts in TILs stratified by patients
UMAP: uniform manifold approximation and projection; TILs: tumor-infiltrating lymphocyte; Sq: squamous cell carcinoma; Ad: adenocarcinoma.

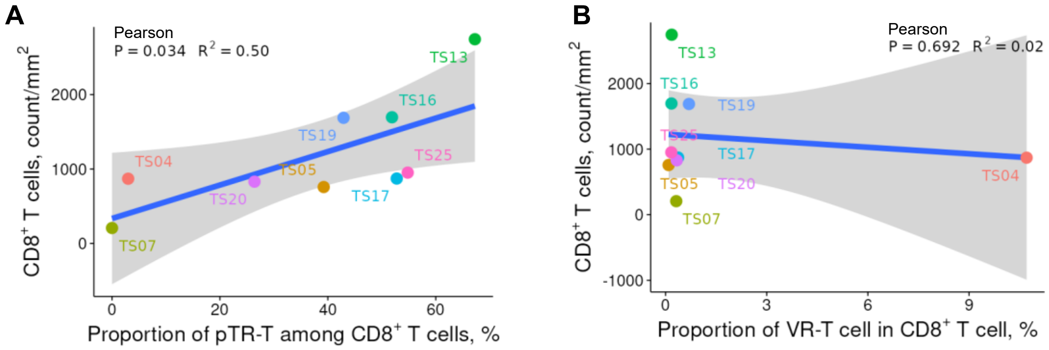

Supplementary Figure 2. Correlation between proportion of pTR-Ts and VR-T and CD8⁺ T cell infiltration in TILs

(A) Proportion of pTR-Ts among total $CD8^+$ TILs is positively associated with total $CD8^+$ T cell infiltration in TILs assessed by pathology specimens.

(B) Proportion of viral-reactive T cells (VR-Ts) among total $CD8^+$ TILs is not correlated with total $CD8^+$ T cell infiltration in TILs assessed by pathology specimens.

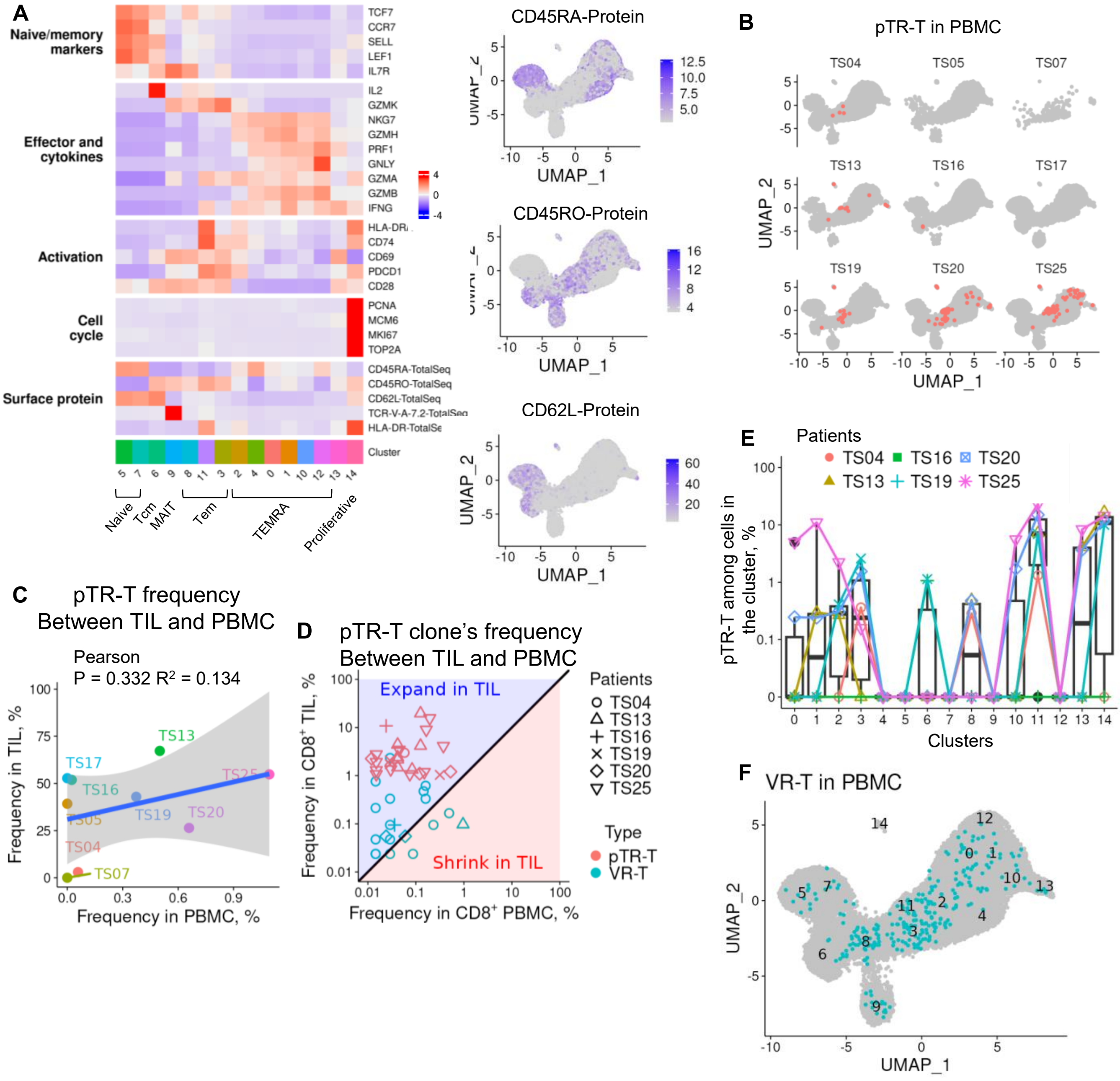

Supplementary Figure 3. Detection of pTR-Ts in PBMCs

(A) Heatmap of cluster-defining gene and surface protein expression (left). Expression of cluster-defining proteins on UMAP (right). MAIT, mucosal-associated invariant T cell; Tcm, central memory T cell; Tem, effector memory T cell; TEMRA, terminally differentiated effector memory T cells re-expressing CD45RA

(B) Distribution of pTR-Ts in PBMCs stratified by patients

(C) Comparison of the frequency of pTR-Ts in TIL and PBMC

(D) pTR-T and VR-T clone frequency between PBMC and TIL. Only clones detected in both TIL and PBMC were plotted

(E) For peripheral $CD8^+$ T cell analysis, the proportions of pTR-Ts in each cluster were plotted, stratified by patient

(F) Circulating VR-Ts mapped onto the UMAP of peripheral $CD8^+$ T cells.

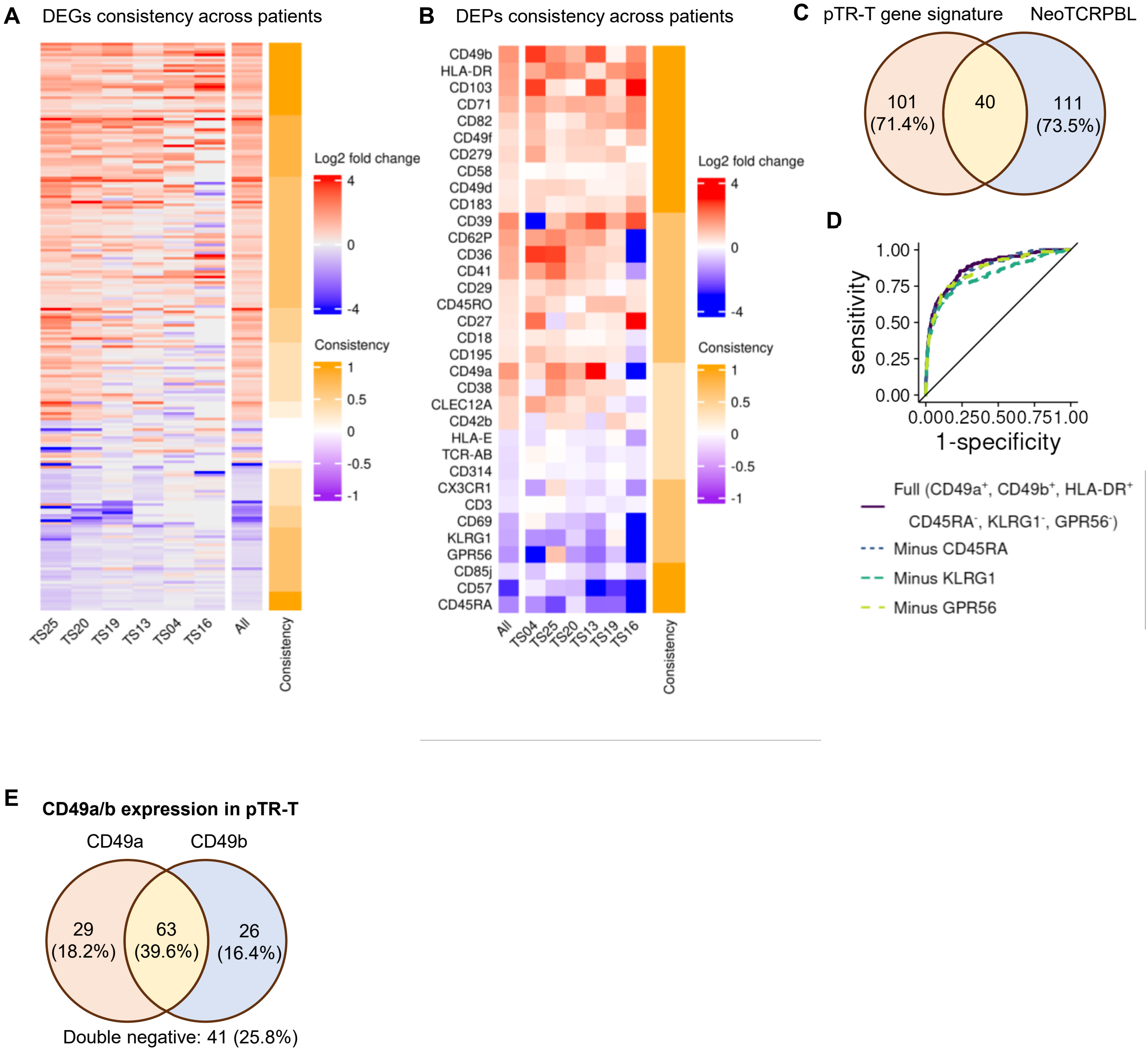

Supplementary Figure 4. Establishment of the pTR-T gene signature and TRTPBL signature

(A) Differentially expressed genes between pTR-Ts and other CD8+ T cells across patients. “All” represents results aggregated from all patient samples, which correspond to the cell-level analysis shown in Figure 4D. Consistency was evaluated based on the direction of $\log_2$ fold change, where a value of 1 indicates that the gene was regulated in the same direction (either up or down) across all patients (see Methods).

(B) Differentially expressed proteins between pTR-Ts and other CD8+ T cells across patients. “All” represents results aggregated from all patient samples, which correspond to the cell-level analysis shown in Figure 4E. Consistency was calculated using the same method described in Supplementary Figure 4A.

(C) Shared and distinct genes between the pTR-T gene signature and the NeoTCRPBL signature [16].

(D) ROC analysis for detecting circulating pTR-Ts based on protein marker combinations. The full marker set including CD49a, CD49b, HLA-DR, CD45RA, GPR56 and KLRG1 is shown as a red line. Combinations excluding CD45RA, KLRG1, and GPR56 are shown as green, blue, and purple dashed lines, respectively.

(E) Expression of CD49a and CD49b in pTR-Ts (159 cells)

DEG: differentially expressed gene; DEP: differentially expressed protein; ROC: receiver operating characteristic

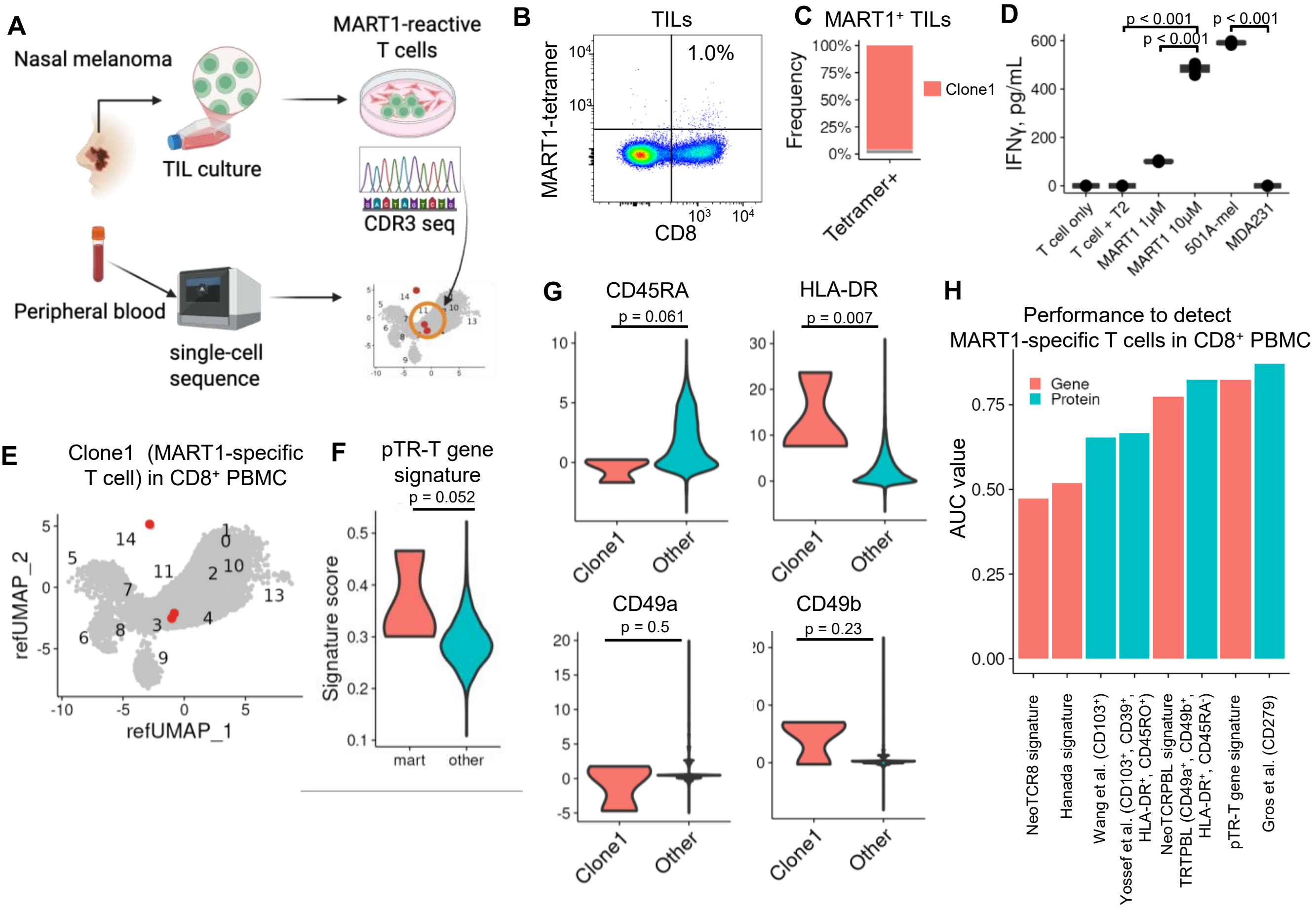

Supplementary Figure 5. Validation of the TRTPBL marker in a melanoma patient

(A) TILs from a melanoma patient were cultured with the MART1 peptide to establish a MART1-specific clone line. In parallel, CD8⁺ TILs and PBMCs from the same patient were subjected to single-cell sequencing. Created in BioRender. Ito, K. (2025) https://BioRender.com/jssrzfr

(B) Frequency of MART1 tetramer-positive cells in the fresh tumor digest

(C) Single-cell TCR sequencing of MART1 tetramer-positive TILs reveals single clone (Clone1) accounted for more than 95% of the population

(D) MART1 specific IFN-γ production from Clone1. Clone 1 was cocultured for 24 hours with T2 cells pulsed with HLA-A*0201-restricted MART1 peptide (MART1 1uM and 10uM) or a MART-1 expressing melanoma cell line (501Amel). IFN-γ levels in the supernatants were measured by ELISA. Negative controls included Clone 1 alone (T cell only), Clone 1 with unpulsed T2 cells (T cell + T2) and Clone 1 with a MART1 negative cell line (MDA231). (technical replicates, n = 3, two-sided unpaired *t*-test)

(E) Clone1 was projected onto in UMAP of CD8⁺ PBMC. Clone1 was located within the cluster 3 and 14.

(F) pTR-T signature scores (Figure 2D and G) of Clone 1 and other CD8⁺ T cells in PBMCs (two-sided unpaired Wilcoxon rank-sum test)

(G) Expression of CD45RA, HLA-DR, CD49a, and CD49b on Clone1 and other CD8⁺ T cells in PBMC (two-sided unpaired Wilcoxon rank-sum test)

(H) Performance comparison of individual markers for detecting MART1-specific T cells within total CD8⁺ T cells in PBMCs. AUC values are shown.

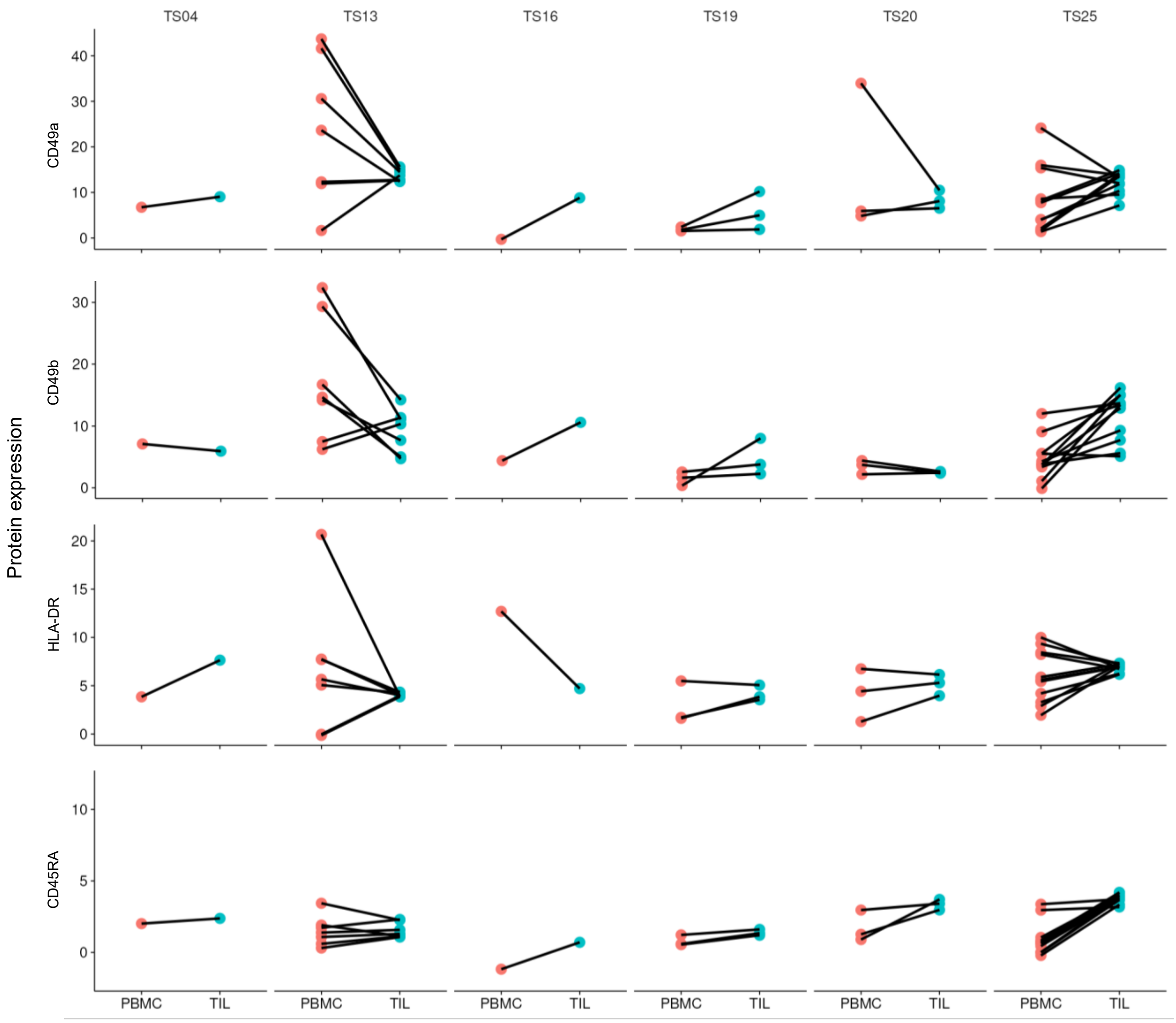

Supplementary Figure 6. TRTPBL markers between pTR-Ts in PBMCs and TILs
Averaged clone-level protein expression of TRTPBL markers (CD49a, CD49b, HLA-DR, and CD45RA) between pTR-Ts in PBMCs and those in TILs stratified by patient. Each dot and line represents a clonotype.

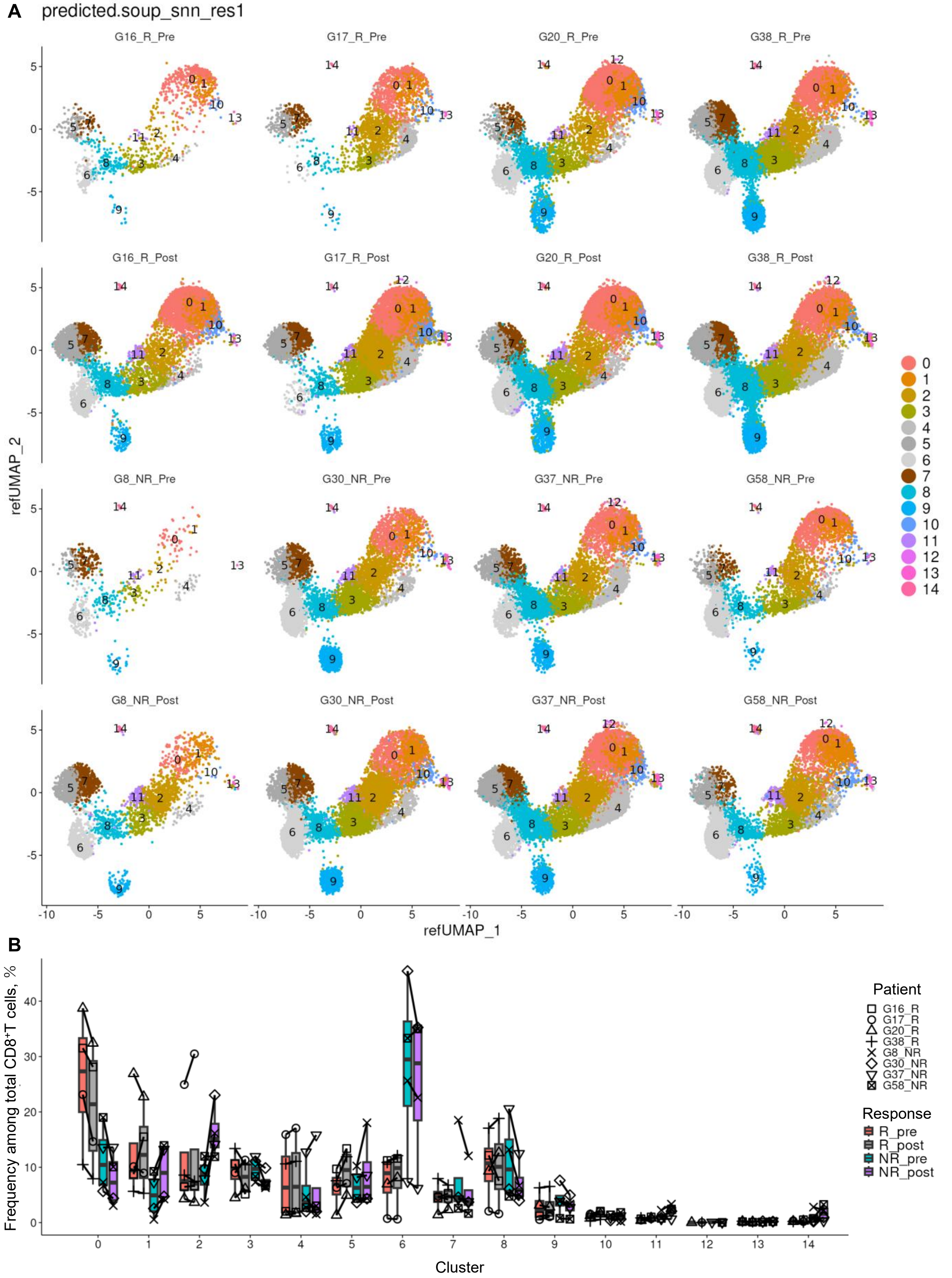

Supplementary Figure 7. Single cell analysis from 8 patients who received ICIs

(A) Pre and post treatment single-cell $CD8^+$ PBMCs from 8 patients who received immune-checkpoint therapy were projected onto the UMAP image (Figure 2A)

(B) Distribution of total $CD8^+$ T cells across clusters in each patient, shown separately by treatment phase (pre- and post-treatment) and response status. In the box plots, the center line indicates the median; the box represents the interquartile range (25th–75th percentiles); whiskers extend to the most extreme values within 1.5 × the interquartile range.

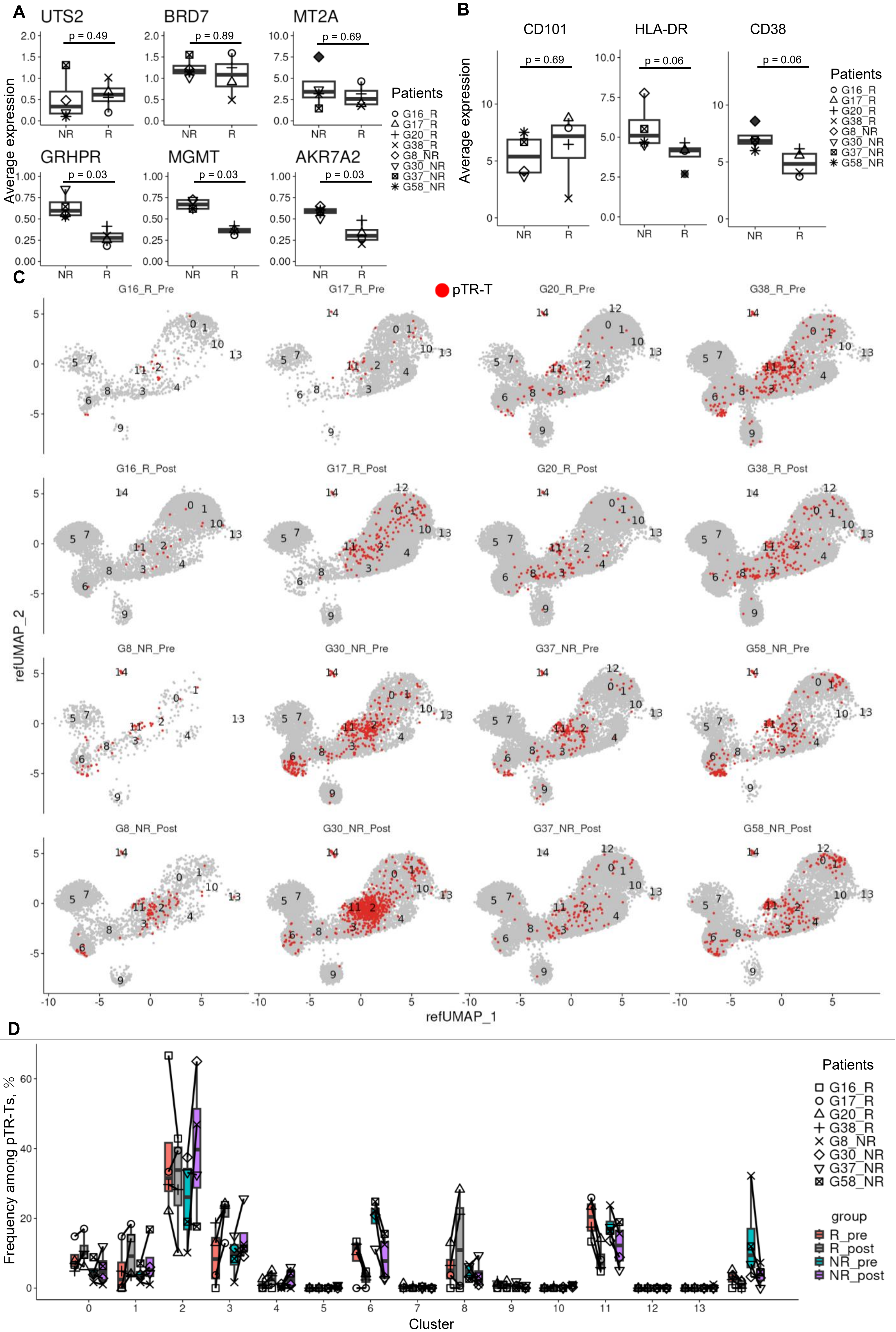

A
UTS2
BRD7
MT2A
p = 0.49
p = 0.89
p = 0.69
GRHPR
MGMT
AKR7A2
p = 0.03
p = 0.03
p = 0.03
Average expression
NR
R
Patients
G16_R
G17_R
G20_R
G38_R
G8_NR
G30_NR
G37_NR
G58_NR
B
CD101
HLA-DR
CD38
p = 0.69
p = 0.06
p = 0.06
Average expression
NR
R
Patients
G16_R
G17_R
G20_R
G38_R
G8_NR
G30_NR
G37_NR
G58_NR
C
pTR-T
G16_R_Pre
G17_R_Pre
G20_R_Pre
G38_R_Pre
G16_R_Post
G17_R_Post
G20_R_Post
G38_R_Post
G8_NR_Pre
G30_NR_Pre
G37_NR_Pre
G58_NR_Pre
G8_NR_Post
G30_NR_Post
G37_NR_Post
G58_NR_Post
refUMAP_2
refUMAP_1
D
Frequency among pTR-Ts, %
Cluster
Patients
G16_R
G17_R
G20_R
G38_R
G8_NR
G30_NR
G37_NR
G58_NR
group
R_pre
R_post
NR_pre
NR_post

Supplementary Figure 8. Expression and differentiation of pTR-Ts between responder and non-responder

(A) Patient-level expression of differentially expressed genes (Figure 4C) between responders' and non-responders' pTR-Ts in pre-treatment samples (unpaired two-sided Wilcoxon rank-sum test).
(B) Patient-level expression of differentially expressed proteins (Figure 4D) between responders' and non-responders' pTR-Ts in pre-treatment samples (unpaired two-sided Wilcoxon rank-sum test).
(C) Patient-level pTR-T distribution shown separately by treatment phase (pre- and post-treatment)
(D) Distribution of pTR-T cells across clusters in each patient, shown separately by treatment phase and response status. The proportion of pTR-T cells within each cluster is displayed for individual patients. In the box plots, the center line indicates the median; the box represents the interquartile range (25th–75th percentiles); whiskers extend to the most extreme values within 1.5 × the interquartile range.

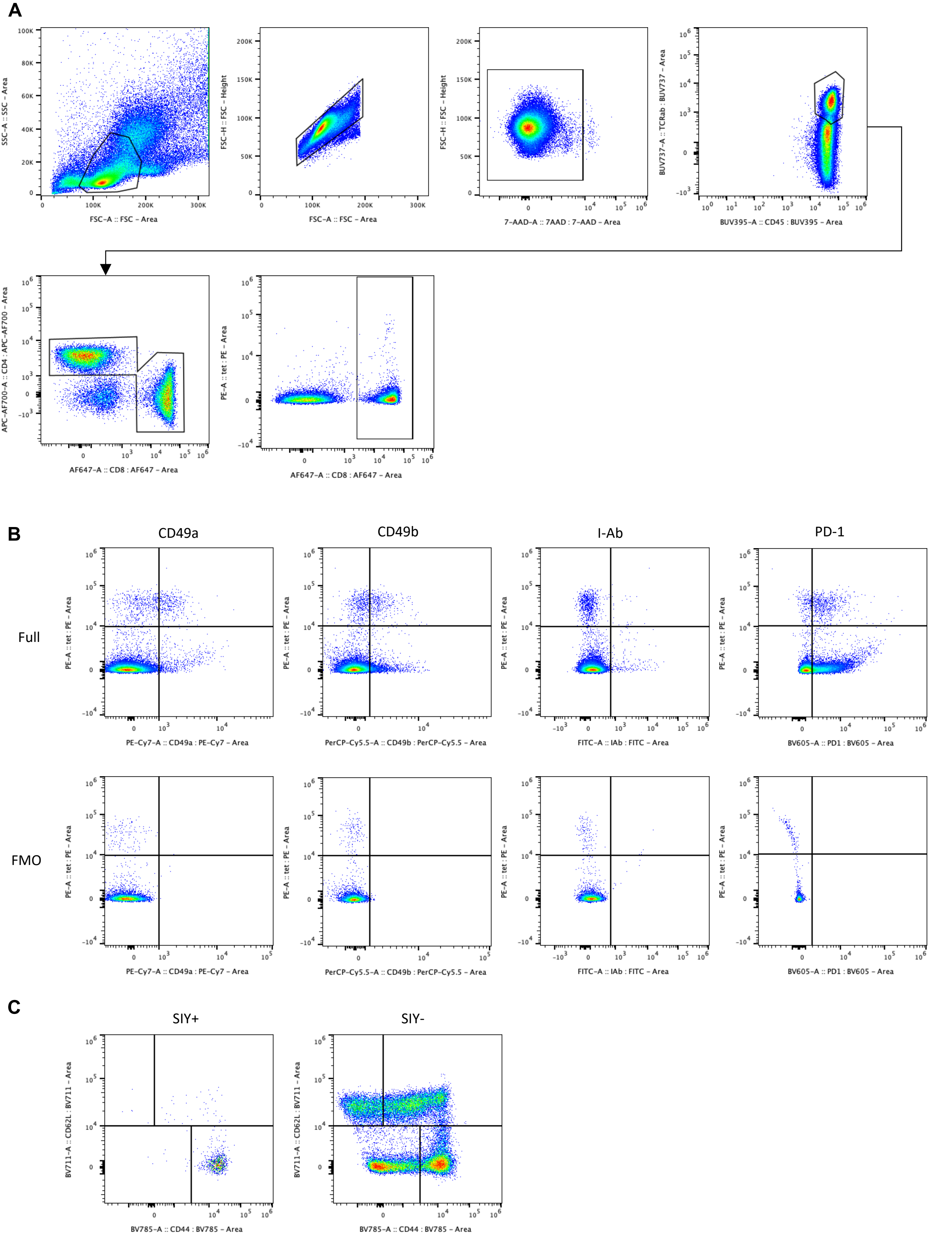

Supplementary Figure 9. Gating strategies used for mouse PBMCs.
(A) Gating strategy to analyze CD8+ T cells in PBMCs from C57BL/6N mice inoculated with a murine melanoma cell line expressing the artificial antigen SIY (BPmel-1-SIY), treated with anti-PD-L1 antibody (80 μg/mouse) on day 7, and sacrificed on day 11
(B) Gating strategy to detect marker-expressed cells in SIY tetramer-positive cells (top). Fluorescent minus one samples (bottom) were used to determine cut-off values, related to Figure 5B
(C) Gating strategy to determine naïve ($CD62L^+$, $CD44^-$), central memory ($CD62L^+$, $CD44^+$), and effector memory ($CD62L^-$, $CD44^+$) cells in SIY tetramer-positive cells (left) and negative cells (right)

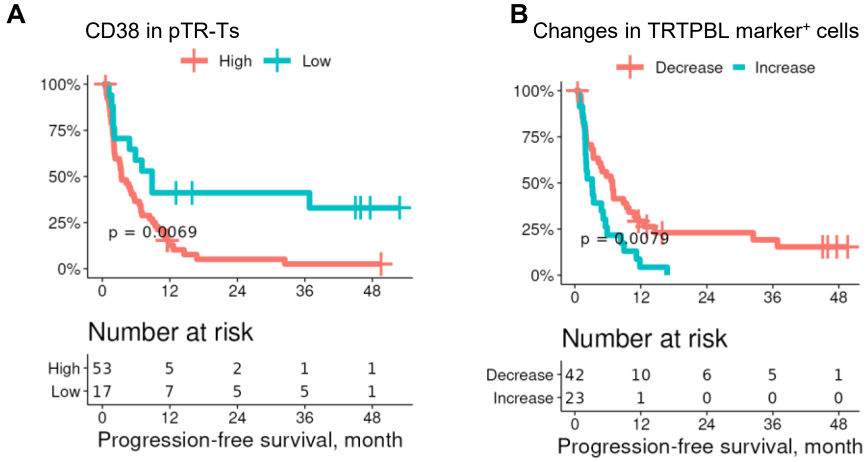

Supplementary Figure 10

(A) Progression-free survival stratified by the proportion of $CD38^+$ cells in TRTPBL $marker^+$ $CD8^+$ T cells (log-rank test)

(B) Progression-free survival stratified by changes in TRTPBL $marker^+$ $CD8^+$ T cells (log-rank test)

Supplementary Table 1. Patient characteristics of paired PBMC and TILs cohort, related to Figure 1

Patient ID	Sex	Age	Histology	Smoking status	Driver mutation	PDL1 TPS	Clinical stage	Pathological stage	Number of cells (TIL)	Number of cells (PBMC)
TS4	M	71	Squamous	Smoker/Ex-smoker	No	NA	cT2aN0M0 StageIB	pT2aN0	4,212	6,895
TS5	M	71	Squamous	Smoker/Ex-smoker	No	1-49%	cT1cN0M0 StageIA3	pT1cN1	2,243	3,368
TS7	M	75	Squamous	Smoker/Ex-smoker	No	1-49%	cT2aN0M0 StageIB	pT2aN1	5,390	200
TS13	M	69	Adenocarcinoma	Smoker/Ex-smoker	No	≥50%	cT2bN0M0 stage IIA	pT3N0	7,181	2,396
TS16	M	79	Adenocarcinoma	Non-smoker	KRAS p.Gly12Ala	<1%	cT2aN0M0 StageIB	pT1cN0	6,335	8,323
TS17	F	76	Squamous	Smoker/Ex-smoker	No	≥50%	cT1bN0M0 StageIA2	pT1bN0	271	6,890
TS19	M	54	Adenocarcinoma	Non-smoker	KRAS p.Gly12Cys	≥50%	cT1cN0M0 StageIA3	pT1cN0	289	3,483
TS20	M	64	Adenocarcinoma	Smoker/Ex-smoker	KRAS p.Gln61His	1-49%	cT1bN0M0 StageIA2	pT1aN0	1,818	8,342
TS25	M	53	Squamous	Smoker/Ex-smoker	No	1-49%	cT3N1M0 StageIIIA	pT3N0	1,770	6,675

Supplementary Table 2. single cell TCR sequence of MART1 tetramer-positive CD8+ TILs, related to supplementary Figure 4C

Clone	CDR3 sequence	Cell count
Clone1	TRB:TGTGCCAGCAGTTTAGCCGGGACTTTAGACTACGAGCAGTACTTC;TRA:TGTGCAGAGGGGCAGGGCGGTACCTCAGGAACCTACAAATACATCTTT;TRA:TGTGCCGTGAACGATGCAGGCAAATCAACCTTT	402
Clone2	TRB:TGTGCCAGCAGCCAAGCTTCAGGGGAGGGCTACAATGAGCAGTTCTTC;TRA:TGTGCAATGAGAGACTTAAATGCTGGTGGTACTAGCTATGGAAAGCTGACATTT	4
Clone3	TRB:TGTGCCCCACCGGGTCTAGCGGAAACTTACGAGCAGTACTTC;TRB:TGTGCCAGCAGTCCATACGGGGGACAGGGAACCGAGCAGTACTTC;TRA:TGTGCATCGGGGGTAGGCTTTGGGAATGTGCTGCATTGC	2
Clone4	TRB:TGCAGTGCAACAACCGGGAACTATGGCTACACCTTC;TRA:TGTGCTACCTATGGGGTATCTAACTTTGGAAATGAGAAATTAACCTTT	1
Clone5	TRA:TGTGCCGTGAACATAAGGATGGACATGCGCTTT	1
Clone6	TRB:TGTGCCACCGGGACAGGGGGTGGCTACACCTTC;TRA:TGTGCAGCAAAAGGAACCGGCACTGCCAGTAAACTCACCTTT	1
Clone8	TRB:TGTGCCAGCAGCCTCGGGCCCGGTGTTGAAGCTTTCTTT;TRA:TGTGCTGTCGTTCTAGAAACCAGTGGCTCTAGGTTGACCTTT	1
Clone9	TRB:TGCGCCAGCAGCCAAATCGGACTAGCGGACAATGAGCAGTTCTTC	1
Clone10	TRB:TGTGCCAGCAGCCGGATGGGCGGGGTGGCCGGGGAGCTGTTTTTT	1
Clone11	TRB:TGTGCCATCAGTGAGTCGAGGTCTTCGGCCGGCGAGCAGTACTTC;TRA:TGTGTGGTGAACATGAGGTTGGAGAGCAGTGCTTCCAAGATAATCTTT	1
Clone12	TRB:TGCGCCAGCAGCTTGGCCCAGGGGACAGGCCACTACGAGCAGTACTTC;TRA:TGTGCTGTGAGTGGGTTTGGAAATGAGAAATTAACCTTT	1
Clone13	TRB:TGTGCCAGCAGATTACAGGGGGTTGCAACTAATGAAAAACTGTTTTTT;TRA:TGTGTGGTGAACCTACCTATATACAACTTCAACAAATTTTACTTT	1
Clone14	TRB:TGTGCCAGCAAAACCTTGGGGGCCTTGGACACCGGGGAGCTGTTTTTT;TRA:TGTGCCGTGAAGGGGGGCCAGAAGCTGCTCTTT	1
Clone15	TRB:TGTGCCAGCAGTTCCCCCCGTACTAGCGGGATATCCTACGAGCAGTACTTC;TRA:TGTGCCGTGAATGCGAGCCAGGCAGGAACTGCTCTGATCTTT	1

Supplementary Table 3. Patient characteristics of paired PBMC and TILs cohort, related to Figure 4.

ID	Sex	Age at sample collection	Histology	Smoking status	Driver mutation	PDL1 TPS	Clinical stage	Treatment	Best objective response	Response	Progression-free survival, days	Cell number
G16	M	73	Adenocarcinoma	Smoker/Ex-smoker	No	≥50%	cT3N0M1c StageIVB	CBDCA+Pemetrexed+Pembrolizumab	Partial response	Responder	714	Pre: 1177<br>Post: 8651
G17	M	66	Adenocarcinoma	Smoker/Ex-smoker	No	<1%	cT1aN2M0 StageIIIA	CBDCA+Paclitaxel+Bevacizumab+Atezolizumab	Partial response	Responder	742	Pre: 2767<br>Post: 17050
G20	M	72	Adenocarcinoma	Smoker/Ex-smoker	No	1-49%	cT4N0M0 StageIIIB	CBDCA+Pemetrexed+Atezolizumab	Partial response	Responder	350	Pre: 12381<br>Post: 24389
G38	M	52	Adenocarcinoma	Smoker/Ex-smoker	No	1-49%	cT2bN1M1a StageIVA	CBDCA+Pemetrexed+Atezolizumab	Partial response	Responder	299	Pre: 10583<br>Post: 15621
G8	M	73	Adenocarcinoma	Smoker/Ex-smoker	No	1-49%	cT1cN0M1c StageIVB	CBDCA+Pemetrexed+Atezolizumab	Stable disease	Non-responder	147	Pre: 714<br>Post: 3843
G30	M	76	Adenocarcinoma	Smoker/Ex-smoker	No	<1%	cT2aN3M1a StageIVA	CBDCA+Pemetrexed+Atezolizumab	Stable disease	Non-responder	130	Pre: 9266<br>Post: 16358
G37	M	50	Adenocarcinoma	Smoker/Ex-smoker	No	1-49%	cT3N2M1c StageIVB	CBDCA+Paclitaxel+Bevacizumab+Atezolizumab	Progressive disease	Non-responder	42	Pre: 11779<br>Post: 19492
G58	M	71	Adenocarcinoma	Smoker/Ex-smoker	No	1-49%	cT1bN0M1a StageIVA	CBDCA+Abraxane+Atezolizumab	Stable disease	Non-responder	170	Pre: 5827<br>Post: 11475

Supplementary Table 4. Patient characteristics of validation cohort, related to Figure 6.

Variables		
Age		66.9 ± 11.4
Sex	Male	49 (70.0)
	Female	21 (30.0)
Smoking history	Current/past	54 (77.1)
	Non-smoker	16 (22.9)
Histology	Adenocarcinoma	54 (77.1)
	Squamous cell carcinoma	16 (22.9)
PDL1 TPS	≥50%	21 (30.0)
	1-49%	19 (27.1)
	<1%	20 (28.6)
	Unknown	10 (14.3)
Treatment	Chemotherapy + anti-PD1/PDL1 antibody	39 (55.7)
	Anti-PD1/PDL1 antibody (monotherapy)	21 (30.0)
	Anti-PD1/PDL1 antibody +anti-CTLA4 antibody ±chemotherapy	10 (14.3)

Data are presented as mean ± SD or number (%).
