# Supplementary material for: Phenotype of circulating tumor-reactive T cells predicts immune checkpoint inhibitor response in non-small cell lung cancer
Source: Nat Commun. 2026 Feb 17;17:2856. doi: 10.1038/s41467-026-69680-x (PMC13022356; doi:10.1038/s41467-026-69680-x)
Supplement: Supplementary file 2 — Description of Additional Supplementary Files [file 41467_2026_69680_MOESM2_ESM.pdf]

Description of Additional Supplementary Files

Supplementary Data 1. Gene signatures used in this study.

Supplementary Data 2. Differentially expressed genes between cluster 11 and the others, related to Figure 2A. P values were adjusted for multiple comparisons using the Benjamini–Hochberg method.

Supplementary Data 3. Differentially expressed genes between circulating TR-T and non-TR-T, related to Figure 2D. P values were adjusted for multiple comparisons using the Benjamini–Hochberg method.

Supplementary Data 4. Differentially expressed proteins between circulating TR-T and non-TR-T, related to Figure 2E. P values were adjusted for multiple comparisons using the Benjamini–Hochberg method.

Supplementary Data 5. Patient-level average log2 fold change of differentially expressed genes between circulating TR-T and non-TR-T, related to Supplementary Figure 4A.

Supplementary Data 6. Patient-level average log2 fold change of differentially expressed proteins between circulating TR-T and non-TR-T, related to Supplementary Figure 4A.

Supplementary Data 7. Differentially expressed genes of circulating pTR-Ts between responder and non-responder, related to Figure 4C. P values were adjusted for multiple comparisons using the Benjamini–Hochberg method.

Supplementary Data 8. Differentially expressed proteins of circulating pTR-Ts between responder and non-responder, related to Figure 4D. P values were adjusted for multiple comparisons using the Benjamini–Hochberg method.
