# Supplementary material for: Phenotype of circulating tumor-reactive T cells predicts immune checkpoint inhibitor response in non-small cell lung cancer
Source: Nat Commun. 2026 Feb 17;17:2856. doi: 10.1038/s41467-026-69680-x (PMC13022356; doi:10.1038/s41467-026-69680-x)
Supplement: Supplementary file 4 — Reporting Summary [file 41467_2026_69680_MOESM4_ESM.pdf]

nature portfolio

Corresponding author(s): Tomonori Yaguchi

Last updated by author(s): Oct 1, 2025

Reporting Summary

Nature Portfolio wishes to improve the reproducibility of the work that we publish. This form provides structure for consistency and transparency in reporting. For further information on Nature Portfolio policies, see our Editorial Policies and the Editorial Policy Checklist.

Statistics

For all statistical analyses, confirm that the following items are present in the figure legend, table legend, main text, or Methods section.

n/a	Confirmed	
☐	☒	The exact sample size (*n*) for each experimental group/condition, given as a discrete number and unit of measurement
☐	☒	A statement on whether measurements were taken from distinct samples or whether the same sample was measured repeatedly
☐	☒	The statistical test(s) used AND whether they are one- or two-sided<br>*Only common tests should be described solely by name; describe more complex techniques in the Methods section.*
☐	☒	A description of all covariates tested
☐	☒	A description of any assumptions or corrections, such as tests of normality and adjustment for multiple comparisons
☐	☒	A full description of the statistical parameters including central tendency (e.g. means) or other basic estimates (e.g. regression coefficient) AND variation (e.g. standard deviation) or associated estimates of uncertainty (e.g. confidence intervals)
☐	☒	For null hypothesis testing, the test statistic (e.g. *F*, *t*, *r*) with confidence intervals, effect sizes, degrees of freedom and *P* value noted<br>*Give P values as exact values whenever suitable.*
☒	☐	For Bayesian analysis, information on the choice of priors and Markov chain Monte Carlo settings
☒	☐	For hierarchical and complex designs, identification of the appropriate level for tests and full reporting of outcomes
☐	☒	Estimates of effect sizes (e.g. Cohen's *d*, Pearson's *r*), indicating how they were calculated

Our web collection on statistics for biologists contains articles on many of the points above.

Software and code

Policy information about availability of computer code

Data collection

All data collection are based on a manufacture's existing protocol or existing method. See "Methods."

Data analysis

Scripts supporting the data analysis were based on existing computational tools and are available in DOI: 10.5281/zenodo.17341751

All softwares and libraries used for the data analysis are as follows.

Single cell data processing: Cell Ranger (v7.0.1, 10x Genomics)

Single cell data analysis:
R version 4.2.2 Patched (2022-11-10 r83330) Platform: x86_64-pc-linux-gnu (64-bit) Running under: Ubuntu 20.04.5 LTS

Matrix products: default BLAS: /usr/lib/x86_64-linux-gnu/blas/libblas.so.3.9.0 LAPACK: /usr/lib/x86_64-linux-gnu/lapack/liblapack.so.3.9.0

locale: [1] LC_CTYPE=C.UTF-8 LC_NUMERIC=C LC_TIME=C.UTF-8 LC_COLLATE=C.UTF-8 LC_MONETARY=C.UTF-8 LC_MESSAGES=C.UTF-8
LC_PAPER=C.UTF-8
[8] LC_NAME=C LC_ADDRESS=C LC_TELEPHONE=C LC_MEASUREMENT=C.UTF-8 LC_IDENTIFICATION=C

attached base packages: [1] grid stats4 stats graphics grDevices utils datasets methods base

other attached packages: [1] dsb_1.0.3 scDblFinder_1.12.0 SoupX_1.6.2 DIALOGUE_1.0 UpSetR_1.4.0
[6] beanplot_1.3.1 Hmisc_5.2-3 ppcor_1.1 reshape2_1.4.4 unikn_1.0.0

[11] RColorBrewer_1.1-3 stringi_1.8.4 psych_2.4.3 plyr_1.8.9 PMA_1.2-4
[16] lmerTest_3.1-3 lme4_1.1-35.3 cowplot_1.1.3 ComplexUpset_1.3.3 preprocessCore_1.60.2
[21] Scissor_2.0.0 Matrix_1.5-1 circlize_0.4.16 ggpubfigs_0.0.1 smotefamily_1.4.0
[26] rpart.plot_3.1.2 rpart_4.1.19 MASS_7.3-58 patchwork_1.2.0.9000 survivalROC_1.0.3.1
[31] pROC_1.18.5 ComplexHeatmap_2.14.0 readxl_1.4.3 survival_3.6-4 survminer_0.4.9
[36] ggpubr_0.6.0 shiny_1.8.1.1 ggridges_0.5.6 SeuratWrappers_0.3.1 monocle3_1.3.1
[41] SingleCellExperiment_1.20.1 SummarizedExperiment_1.28.0 GenomicRanges_1.50.2 GenomeInfoDb_1.34.9 IRanges_2.32.0
[46] S4Vectors_0.36.2 MatrixGenerics_1.10.0 matrixStats_1.3.0 Biobase_2.58.0 BiocGenerics_0.44.0
[51] clusterProfiler_4.6.2 ggalluvial_0.12.5 ggrepel_0.9.5 ggpmisc_0.6.0 ggpp_0.5.8-1
[56] UCell_2.2.0 harmony_1.2.0 Rcpp_1.0.12 scales_1.3.0 lubridate_1.9.3
[61] forcats_1.0.0 stringr_1.5.1 dplyr_1.1.4 purrr_1.0.2 readr_2.1.5
[66] tidyr_1.3.1 tibble_3.2.1 ggplot2_3.5.1 tidyverse_2.0.0 SeuratObject_4.1.3
[71] Seurat_4.3.0.1

IHC analysis: QuPath (version 0.2.3)

Flow cytometer analysis: FlowJo v10

For manuscripts utilizing custom algorithms or software that are central to the research but not yet described in published literature, software must be made available to editors and reviewers. We strongly encourage code deposition in a community repository (e.g. GitHub). See the Nature Portfolio guidelines for submitting code & software for further information.

Data

Policy information about availability of data

All manuscripts must include a data availability statement. This statement should provide the following information, where applicable:

- Accession codes, unique identifiers, or web links for publicly available datasets
- A description of any restrictions on data availability
- For clinical datasets or third party data, please ensure that the statement adheres to our policy

The single-cell RNA sequencing data generated in this study have been deposited in the NCBI Sequence Read Archive (SRA) under the BioProject accession number PRJNA1415239. The single cell data generated in this study and all source data supporting the findings of this study have been deposited in the Zenodo database (https://doi.org/10.5281/zenodo.17342100). The raw sequence data are protected and are available from the corresponding author upon reasonable request and ethical approval due to the consent from patients.

Research involving human participants, their data, or biological material

Policy information about studies with human participants or human data. See also policy information about sex, gender (identity/presentation), and sexual orientation and race, ethnicity and racism.

Reporting on sex and gender	All human studies included both male and female participants, unless otherwise stated. For the patient cohort with NSCLC, sex was recorded and used for sample stratification. Assigned sex determined by national health insurance was used for the analysis.
Reporting on race, ethnicity, or other socially relevant groupings	No socially constructed or relevant categorization variables was used in this study.
Population characteristics	We enrolled 9 patients with surgically removed NSCLC, one patients with melanoma, and 70 patients with metastatic NSCLC. all covariate related to variables were reported in Supplementary Tables.
Recruitment	We recruited patients if the informed consent was obtained from the participant. No compensation was made for participation. For single cell experiment, samples were chosen based on availability and numbers of cells obtained. For flow cytometry data, all patients who meet the inclusion criteria were analyzed.
Ethics oversight	Human studies were approved by the institutional review board of Kyoto University Graduate School and Faculty of Medicine (approval number G1012).

Note that full information on the approval of the study protocol must also be provided in the manuscript.

Field-specific reporting

Please select the one below that is the best fit for your research. If you are not sure, read the appropriate sections before making your selection.

☒ Life sciences ☐ Behavioural & social sciences ☐ Ecological, evolutionary & environmental sciences

For a reference copy of the document with all sections, see nature.com/documents/nr-reporting-summary-flat.pdf

Life sciences study design

All studies must disclose on these points even when the disclosure is negative.

Sample size	For human study, no pre-defined sample number was used due to the exploratory nature of the study and availability of samples.<br>For mouse study, a group size of 10 animals per condition was chosen based on general practice in the field and our prior experience.
Data exclusions	No data were excluded from the analysis.
Replication	Mouse experiment was repeated one time (Figure 5). Data were consistent across experiments. No replication experiment was done for human materials due to the sample limitation.
Randomization	Mouse are randomly allocated to treated or control group after tumor inoculation.
Blinding	Blinding was not performed during animal experiments and in vitro experiments due to the nature of the experimental procedures. Blinding was not relevant for computational analyses, as data processing and analysis were performed using automated and objective methods.

Reporting for specific materials, systems and methods

We require information from authors about some types of materials, experimental systems and methods used in many studies. Here, indicate whether each material, system or method listed is relevant to your study. If you are not sure if a list item applies to your research, read the appropriate section before selecting a response.

Materials & experimental systems

n/a	Involved in the study
☐	☒ Antibodies
☐	☒ Eukaryotic cell lines
☐	☐ Palaeontology and archaeology
☐	☒ Animals and other organisms
☐	☒ Clinical data
☐	☐ Dual use research of concern
☐	☐ Plants

Methods

n/a	Involved in the study
☐	☐ ChIP-seq
☐	☒ Flow cytometry
☐	☐ MRI-based neuroimaging

Antibodies

Antibodies used

Antibodies for cell sorting
7-AAD (TONBO Biosciences)
CD45 PE (2D1, BioLegend)
CD8 APC (PRA-T8, BioLegend)

Flow cytometry panel for mouse
CD45 BUV395 (30-F11, BD Horizon)
TCRβ BUV737 (H57-597, BD Horizon)
CD8 AF647 (KT15, MBL)
CD4 AF700 (RM4-5, BioLegend)
CD49a PE-Cy7 (HMa1, BioLegend)
CD49b PerCP/Cy5.5 (HMa1, BioLegend)
PD-1 BV605 (29F.1A12, BioLegend)
MHC-II (I-A/I-E) FITC (M5/114.15.2; BioLegend)
CD62L BV711 (MEL-14, BioLegend)
CD44 BV785 (IM7, BioLegend)
7-AAD (TOMBO Biosciences)

Flow cytometry for human
TCRα/β BUV737 (IP26, BD OptiBuild)
CD8 BUV395 (RPA-T8, BD Horizon)
CD4 APC-Cy7 (RPA-T4, TONBO Biosciences)
CD49a PE-Cy7 (TS2/7, BioLegend)
CD49b FITC (P1E6-C5, BioLegend)
CD45RA vF450 (HI100, TONBO Biosciences)
HLA-DR PE/Dazzle 594 (L243, BioLegend)
CD38 BV711 (HIT2, BioLegend)
7-AAD (TONBO Biosciences)

Antibody used for mouse experiment
anti–PD-L1 monoclonal antibody (clone 1-111A.4)

	Isotype control (rat IgG2a, κ; Bio X Cell)<br><br>Immunohistochemical staining<br>anti-CD8 antibody (clone SP57, VENTANA)
Validation	All flowcytometry and immunohistochemical staining antibody was validated by vendors.<br>Anti-PD-L1 monoclonal antibody was validated previously. (M. Ishida, Y. Iwai, Y. Tanaka, T. Okazaki, G. J. Freeman, N. Minato, T. Honjo, Differential expression of PD-L1 and PD-L2, ligands for an inhibitory receptor PD-1, in the cells of lymphohematopoietic tissues. Immunol. Lett. 84, 57–62 (2002).)

Eukaryotic cell lines

Policy information about cell lines and Sex and Gender in Research

Cell line source(s)	BPmel-1-SIY was established in our laboratory as previously reported.
Authentication	None of the cell lines are authenticated
Mycoplasma contamination	All cell lines tested negative for mycoplasma contamination.
Commonly misidentified lines<br>(See ICLAC register)	No commonly misidentified lines was used.

Palaeontology and Archaeology

Specimen provenance	*Provide provenance information for specimens and describe permits that were obtained for the work (including the name of the issuing authority, the date of issue, and any identifying information). Permits should encompass collection and, where applicable, export.*
Specimen deposition	*Indicate where the specimens have been deposited to permit free access by other researchers.*
Dating methods	*If new dates are provided, describe how they were obtained (e.g. collection, storage, sample pretreatment and measurement), where they were obtained (i.e. lab name), the calibration program and the protocol for quality assurance OR state that no new dates are provided.*

☐ Tick this box to confirm that the raw and calibrated dates are available in the paper or in Supplementary Information.

Ethics oversight	*Identify the organization(s) that approved or provided guidance on the study protocol, OR state that no ethical approval or guidance was required and explain why not.*

Note that full information on the approval of the study protocol must also be provided in the manuscript.

Animals and other research organisms

Policy information about studies involving animals; ARRIVE guidelines recommended for reporting animal research, and Sex and Gender in Research

Laboratory animals	Six-week-old female C57BL/6N mice are obtained from Clea Japan.
Wild animals	No wild animal was used.
Reporting on sex	Mouse experiments used female C57BL/6N mice for consistency with prior tumor models and to reduce hormonal variability. These findings are expected to be relevant for both sex.
Field-collected samples	No field-collected sample was used.
Ethics oversight	All animal experiments were approved by the Animal Research Committee of Kyoto University and were performed in accordance with institutional guidelines.

Note that full information on the approval of the study protocol must also be provided in the manuscript.

Clinical data

Policy information about clinical studies

All manuscripts should comply with the ICMJE guidelines for publication of clinical research and a completed CONSORT checklist must be included with all submissions.

Clinical trial registration	*Provide the trial registration number from ClinicalTrials.gov or an equivalent agency.*
Study protocol	*Note where the full trial protocol can be accessed OR if not available, explain why.*

Data collection	*Describe the settings and locales of data collection, noting the time periods of recruitment and data collection.*
Outcomes	*Describe how you pre-defined primary and secondary outcome measures and how you assessed these measures.*

Dual use research of concern

Policy information about dual use research of concern

Hazards

Could the accidental, deliberate or reckless misuse of agents or technologies generated in the work, or the application of information presented in the manuscript, pose a threat to:

No	Yes	
☒	☐	Public health
☒	☐	National security
☒	☐	Crops and/or livestock
☒	☐	Ecosystems
☒	☐	Any other significant area

Experiments of concern

Does the work involve any of these experiments of concern:

No	Yes	
☒	☐	Demonstrate how to render a vaccine ineffective
☒	☐	Confer resistance to therapeutically useful antibiotics or antiviral agents
☒	☐	Enhance the virulence of a pathogen or render a nonpathogen virulent
☒	☐	Increase transmissibility of a pathogen
☒	☐	Alter the host range of a pathogen
☒	☐	Enable evasion of diagnostic/detection modalities
☒	☐	Enable the weaponization of a biological agent or toxin
☒	☐	Any other potentially harmful combination of experiments and agents

Plants

Seed stocks	*Report on the source of all seed stocks or other plant material used. If applicable, state the seed stock centre and catalogue number. If plant specimens were collected from the field, describe the collection location, date and sampling procedures.*
Novel plant genotypes	*Describe the methods by which all novel plant genotypes were produced. This includes those generated by transgenic approaches, gene editing, chemical/radiation-based mutagenesis and hybridization. For transgenic lines, describe the transformation method, the number of independent lines analyzed and the generation upon which experiments were performed. For gene-edited lines, describe the editor used, the endogenous sequence targeted for editing, the targeting guide RNA sequence (if applicable) and how the editor was applied.*
Authentication	*Describe any authentication procedures for each seed stock used or novel genotype generated. Describe any experiments used to assess the effect of a mutation and, where applicable, how potential secondary effects (e.g. second site T-DNA insertions, mosiacism, off-target gene editing) were examined.*

ChIP-seq

Data deposition

☐ Confirm that both raw and final processed data have been deposited in a public database such as GEO.

☐ Confirm that you have deposited or provided access to graph files (e.g. BED files) for the called peaks.

Data access links<br>*May remain private before publication.*	*For "Initial submission" or "Revised version" documents, provide reviewer access links. For your "Final submission" document, provide a link to the deposited data.*
Files in database submission	*Provide a list of all files available in the database submission.*
Genome browser session<br>(e.g. UCSC)	*Provide a link to an anonymized genome browser session for "Initial submission" and "Revised version" documents only, to enable peer review. Write "no longer applicable" for "Final submission" documents.*

Methodology

Replicates	*Describe the experimental replicates, specifying number, type and replicate agreement.*
Sequencing depth	*Describe the sequencing depth for each experiment, providing the total number of reads, uniquely mapped reads, length of reads and whether they were paired- or single-end.*
Antibodies	*Describe the antibodies used for the ChIP-seq experiments; as applicable, provide supplier name, catalog number, clone name, and lot number.*
Peak calling parameters	*Specify the command line program and parameters used for read mapping and peak calling, including the ChIP, control and index files used.*
Data quality	*Describe the methods used to ensure data quality in full detail, including how many peaks are at FDR 5% and above 5-fold enrichment.*
Software	*Describe the software used to collect and analyze the ChIP-seq data. For custom code that has been deposited into a community repository, provide accession details.*

Flow Cytometry

Plots

Confirm that:

☒ The axis labels state the marker and fluorochrome used (e.g. CD4-FITC).

☒ The axis scales are clearly visible. Include numbers along axes only for bottom left plot of group (a 'group' is an analysis of identical markers).

☒ All plots are contour plots with outliers or pseudocolor plots.

☒ A numerical value for number of cells or percentage (with statistics) is provided.

Methodology

Sample preparation	For cell sorting for single-cell library preparation, cells were stained with panels on ice for 40 min. Cells were washed using the Curiox system (Tomy Digital Biology) at 10 µL/s × 7 cycles and underwent cell sorting. PBS containing 2% fetal calf serum (FCS) were used for staining and washing.<br>For mouse PBMC staining, Peripheral blood (100 µL) was collected from the mice and lysed using 1 mL of VersaLyse buffer (Beckman Coulter) for 10 min at room temperature. Cells were washed twice with PBS containing 2% FCS and blocked with 1% anti-CD16/32 Fc blocker (93, BioLegend) in 100µL. SIY-specific $CD8^+$ T cells were stained with SIY tetramer (T-Select H-2Kb Negative (SIY) Tetramer, MBL, 2.5% v/v) and incubated for 20 min at room temperature. After washing and diluting in Brilliant Stain Buffer (BD Biosciences), the cells were stained with the following fluorochrome-conjugated antibodies for 20 min at room temperature. Stained samples were washed twice with 2% FCS/PBS and analyzed using an ID7000 spectral flow cytometer.<br>For human PBMC staining, 1×10^5 PBMCs were incubated with human IgG (FUJIFILM) as an Fc blocker (1% v/v) for 30 min at 4 °C, followed by adding the antibody cocktail without washing.Stained samples were washed twice with 2% FCS/PBS and analyzed using an ID7000 spectral flow cytometer.
Instrument	Flow cytometry: ID7000 spectral flow cytometer (Sony Biotechnology)<br>Cell sorting: FACS Melody (BD Biosciences)
Software	Flowjo V10 software was used for analysis.
Cell population abundance	For cell sorting for single-cell library preparation, purity is > 90% for all samples.
Gating strategy	Cells are gated based on FSC/SSC, singlet, and live/dead markers. All cells which showed positive for CD45, CD8,and TCRbeta for mouse experiment and CD8, and TCRalpha/beta were considered as CD8+ T cells. Fluorescent minus one staining (panel except for one color) were prepared for all colors to determine the cutoff of positive and negative.

☐ Tick this box to confirm that a figure exemplifying the gating strategy is provided in the Supplementary Information.

Magnetic resonance imaging

Experimental design

Design type	*Indicate task or resting state; event-related or block design.*
Design specifications	*Specify the number of blocks, trials or experimental units per session and/or subject, and specify the length of each trial or block (if trials are blocked) and interval between trials.*
Behavioral performance measures	*State number and/or type of variables recorded (e.g. correct button press, response time) and what statistics were used*

Behavioral performance measures | *to establish that the subjects were performing the task as expected (e.g. mean, range, and/or standard deviation across subjects).*

Acquisition

Imaging type(s) | *Specify: functional, structural, diffusion, perfusion.*

Field strength | *Specify in Tesla*

Sequence & imaging parameters | *Specify the pulse sequence type (gradient echo, spin echo, etc.), imaging type (EPI, spiral, etc.), field of view, matrix size, slice thickness, orientation and TE/TR/flip angle.*

Area of acquisition | *State whether a whole brain scan was used OR define the area of acquisition, describing how the region was determined.*

Diffusion MRI ☐ Used ☐ Not used

Preprocessing

Preprocessing software | *Provide detail on software version and revision number and on specific parameters (model/functions, brain extraction, segmentation, smoothing kernel size, etc.).*

Normalization | *If data were normalized/standardized, describe the approach(es): specify linear or non-linear and define image types used for transformation OR indicate that data were not normalized and explain rationale for lack of normalization.*

Normalization template | *Describe the template used for normalization/transformation, specifying subject space or group standardized space (e.g. original Talairach, MNI305, ICBM152) OR indicate that the data were not normalized.*

Noise and artifact removal | *Describe your procedure(s) for artifact and structured noise removal, specifying motion parameters, tissue signals and physiological signals (heart rate, respiration).*

Volume censoring | *Define your software and/or method and criteria for volume censoring, and state the extent of such censoring.*

Statistical modeling & inference

Model type and settings | *Specify type (mass univariate, multivariate, RSA, predictive, etc.) and describe essential details of the model at the first and second levels (e.g. fixed, random or mixed effects; drift or auto-correlation).*

Effect(s) tested | *Define precise effect in terms of the task or stimulus conditions instead of psychological concepts and indicate whether ANOVA or factorial designs were used.*

Specify type of analysis: ☐ Whole brain ☐ ROI-based ☐ Both

Statistic type for inference | *Specify voxel-wise or cluster-wise and report all relevant parameters for cluster-wise methods.*

(See Eklund et al. 2016)

Correction | *Describe the type of correction and how it is obtained for multiple comparisons (e.g. FWE, FDR, permutation or Monte Carlo).*

Models & analysis

n/a	Involved in the study
☐	☐ Functional and/or effective connectivity
☐	☐ Graph analysis
☐	☐ Multivariate modeling or predictive analysis

Functional and/or effective connectivity | *Report the measures of dependence used and the model details (e.g. Pearson correlation, partial correlation, mutual information).*

Graph analysis | *Report the dependent variable and connectivity measure, specifying weighted graph or binarized graph, subject- or group-level, and the global and/or node summaries used (e.g. clustering coefficient, efficiency, etc.).*

Multivariate modeling and predictive analysis | *Specify independent variables, features extraction and dimension reduction, model, training and evaluation metrics.*
